# Real-Time Three-Dimensional Echocardiography: Characterization of Cardiac Anatomy and Function—Current Clinical Applications and Literature Review Update

**DOI:** 10.1089/biores.2016.0033

**Published:** 2017-03-01

**Authors:** Omar Velasco, Morgan Q. Beckett, Aaron W. James, Megan N. Loehr, Taylor G. Lewis, Tahmin Hassan, Rajesh Janardhanan

**Affiliations:** ^1^ Department of Internal Medicine, University of Arizona, Tucson, Arizona.; ^2^ Department of Chemistry and Biochemistry, University of Arizona, Tucson, Arizona.; ^3^ Department of Pathology, Johns Hopkins University, Baltimore, Maryland.; ^4^ Banner University Medical Center, Sarver Heart Center, Tucson, Arizona.

**Keywords:** echocardiogram, four-dimensional, left ventricular function, left ventricular mass, left ventricular volume, real-time, right ventricular volume, stroke volume, three-dimensional, transthoracic, two-dimensional

## Abstract

Our review of real-time three-dimensional echocardiography (RT3DE) discusses the diagnostic utility of RT3DE and provides a comparison with two-dimensional echocardiography (2DE) in clinical cardiology. A Pubmed literature search on RT3DE was performed using the following key words: transthoracic, two-dimensional, three-dimensional, real-time, and left ventricular (LV) function. Articles included perspective clinical studies and meta-analyses in the English language, and focused on the role of RT3DE in human subjects. Application of RT3DE includes analysis of the pericardium, right ventricular (RV) and LV cavities, wall motion, valvular disease, great vessels, congenital anomalies, and traumatic injury, such as myocardial contusion. RT3DE, through a transthoracic echocardiography (TTE), allows for increasingly accurate volume and valve motion assessment, estimated LV ejection fraction, and volume measurements. Chamber motion and LV mass approximation have been more accurately evaluated by RT3DE by improved inclusion of the third dimension and quantification of volumetric movement. Moreover, RT3DE was shown to have no statistical significance when comparing the ejection fractions of RT3DE to cardiac magnetic resonance (CMR). Analysis of RT3DE data sets of the LV endocardial exterior allows for the volume to be directly quantified for specific phases of the cardiac cycle, ranging from end systole to end diastole, eliminating error from wall motion abnormalities and asymmetrical left ventricles. RT3DE through TTE measures cardiac function with superior diagnostic accuracy in predicting LV mass, systolic function, along with LV and RV volume when compared with 2DE with comparable results to CMR.

## Introduction

The newly developed real-time three-dimensional echocardiography (RT3DE) imaging technique aims to noninvasively characterize cardiac anatomy and function for clinical decision-making. This novel technique uses temporal information, along with spatial feature extraction, to compile information of cardiac anatomy and function.^1^ Zhang et al. described the potential utility of quantitative analysis of left ventricular (LV) function for suitability of cardiac resynchronization therapy.^2^ The potential application of RT3DE is similar to two-dimensional echocardiography (2DE) with increased accuracy,^3,4^ which includes analysis of the pericardium, right ventricular (RV) and LV cavities, wall motion, congenital anomalies, and traumatic injury, such as myocardial contusion. In this article, we review current literature regarding the evaluation of RT3DE through a transthoracic echocardiography approach for characterization of cardiac anatomy and function for clinical decision-making.

## How Is RT3DE Used for Cardiac Evaluation?

### Quantification of LV mass using RT3DE

RT3DE has been used in the literature to collect 3D scan volume of the LV cavity.^5^ End diastolic volume, the largest cavity volume in the cardiac cycle, has remained the selected frame for measuring the LV capacity volume and LV epicardial volume. Using the long axis of the LV cavity from apex to base as the selected reference, additional points on each side of the endocardial surface along a 45° rotated axis are selected and combined to form the complete surface of the LV cavity. A partial differential equation is then used to create a 3D surface detection algorithm and direct the surface toward the necessary endocardial position. Next, the end diastolic volume of the surface can be measured to obtain LV cavity volume and LV epicardial volume. Subtracting the LV cavity volume from the LV epicardial volume provides the LV myocardial volume, which is then multiplied by myocardial density to provide LV mass.^6^ Compared with 2DE and cardiac magnetic resonance (CMR) imaging, RT3DE provides the most accurate approximation of the LV mass.^6^

### Estimation and comparison of volume and systolic function of left ventricle by RT3DE versus 2DE

The RT3DE is useful in analyzing the LV volumes, ejection fraction, systolic function, and dyssynchrony.^5^ Steady pacing minimizes the spatial and temporal resolution limitation with one beat analysis in 2DE.^7^ In the literature, the attainability of one beat full volume of LV has been noted to be ∼84%.^8^ Currently, there exist data collection systems that utilize sampled matrix array transducers capable of acquiring volumetric data by 3D analysis of multiplanar measurements of volumes.^7^ A study conducted by Corsi et al. illustrates multiple LV volume time curves depicting its accuracy in volumetric analysis.^9^ Comparing these curves with CMR data of the same patients revealed a high degree of correspondence of r = 0.98 and that were thus determined to be statistically similar. Comparing the LV function of nine dilated cardiomyopathy patients with that of nine control patients further validated this novel imaging modality. The RT3DE results showed that the cardiomyopathy patients had significantly lower LV function than the control with no statistical significant difference between RT3DE and CMR.^10^

### Evaluation of left ventricle ejection fraction by RT3DE

The American Heart Association has referred to the ejection fraction as a measure of blood pumping from the LV with each contraction.^10^ Some investigators, such as Sugeng et al., have compared RT3DE with the cardiac CT technique to estimate the ejection fraction.^10^ Moreover, RT3DE was shown to have no statistical significance when comparing the ejection fractions. The measured regional ejection fraction from RT3DE was also found to be in concordance with those from CMR, with zero statistical bias. This indicates a high level of agreement between RT3DE data and CMR data as a reliable source of LV (volume) function.

### Ability of RT3DE for determination of LV and RV stroke volumes

Errors in the LV volume have been shown to contribute to inaccurate volume estimations when the following deformities are present: wall motion abnormalities, aneurysms, and disproportionate LV and RV cardiac chambers.^11^ Specifically, in RV stroke volume determination, proper evaluation has been hindered because of its innate features, such as its infundibulum, asymmetrical shape, and trabecular character.^11^ Because of the significant structural difference between both ventricles, listed hereunder are the recommended approaches in determining the respective stroke volumes using RT3DE.

## LV Stroke Volume

A common approach used in LV stroke volume determination is the analysis of RT3DE data sets gathered by semiautomated recognition software of the LV endocardial exterior. This allows for the volume calculation within the LV for specific phases of the cardiac cycle, ranging from end systole to end diastole.^12^ Moreover, RT3DE data collection allows for direct quantification of the LV stroke volume. It remains unaffected by foreshortened LV views and unreliant geometric modeling estimations. Thus, error from wall motion abnormalities and asymmetrical left ventricles is eliminated.^12–18^

## RV Stroke Volume

The RV stroke volume determination has proven to be a significant challenge because of its complex chamber geometry. Initially, a lack of RT3DE observation of the RV by the conventional ventricular analysis method resulted in inaccurate estimations.^19,20^ The use of the disk summation technique proved to be an accurate methodology for RV volume determination. The disk summation technique is an in-depth analysis of disk thickness, orientation, and gain setting in the right ventricle.^19,20^ A summation of numerous disks are made that layer the RV endocardial surface to measure the ventricle volume change during the cardiac cycles of end diastole and end systole. With the data sets obtained from RT3DE data sets, the base area of each disk and the vertical axis chamber height are summed to determine RV stroke volume. Of note, ventricular papillary muscle and the cardiac trabeculations are not included in the summation, producing a slight volume overestimation.^21^

## Conclusion

RT3DE provides incremental value for the assessment of intracardiac masses through offering a more precise evaluation of the shape, size, and the mass composition.^22,23^ The improved reproducibility and accuracy of the LV volume and ejection fraction measurements lend precision during cardiac evaluation.^11^ In contrast, 2DE produces planar images with inaccuracies, because of microembolization of image capture from beat to beat by the heart, less experienced in RT3DE. One can evaluate regional wall motion in the entire heart, which can be used in the determination of abnormal wall motion with minimal artifacts.^24^ Unfortunately, RT3DE, like 2DE, has limitations and potential error.

Other potential sources of error described in the literature include phantom imaging, intermodality analysis-related differences, and differences in LV boundary identification, such as inclusion of endocardial trabeculae and mitral valve plane.^25^ Interestingly, the volume estimation by RT3DE was similar to the CMR images, but lower at institutions with lower level of experience.^25^ The RT3DE inconsistent estimation of the ventricular volumes was because of sonographer's inability to recognize the difference between myocardium and trabeculae tissue.^26,27^ Tracing the endocardium so that it includes trabeculae was a solution suggested to minimize inconsistent estimation of ventricular volume by RT3DE.

The apparent limitations of the RT3DE are largely related to its ongoing development,^28,29^ specifically, the low rates of frames caused by imperfect translation of data sets and lack of agreement among the various softwares used for 3D evaluation.^30–33^ Overall, the RT3DE method has improved image quality from the standard 2D images and measured cardiac function with superior diagnostic accuracy in predicting LV mass, systolic function, along with LV and RV volume when compared with 2DE. The results from the regional wall motion abnormalities need to be verified in larger studies.

## Abbreviations Used

2DE: two-dimensional echocardiography
CMR: cardiac magnetic resonance
LV: left ventricular
RT3DE: real-time three-dimensional echocardiography
RV: right ventricular
